# Impact of nutritional source modality on weight loss and BMI reduction after hematopoietic stem cell transplantation

**DOI:** 10.3389/fonc.2026.1778224

**Published:** 2026-02-25

**Authors:** Francesco Paolo Tambaro, Francesco Fabozzi, Riccardo Masetti, Fabiana Cacace, Gennaro Pagano, Francesco Cecere, Valeria Caprioli, Giuseppina De Simone, Maria Rosaria D’Amico, Emanuela Rossitti, Edoardo Muratore, Davide Leardini, Maria Simona Sabbatino

**Affiliations:** 1 Pediatric Oncology, Hematology and Cell Therapy, Azienda Ospedaliera di Rilievo Nazionale (AORN) Santobono Pausilipon, Napoli, Italy; 2 Pediatric Hematology and Oncology, Istituto di Ricerca e Cura a Carattere Scientifico (IRCCS) Azienda Ospedaliero-Universitaria di Bologna, Bologna, Italy; 3 Department of Medical and Surgical Sciences (DIMEC), University of Bologna, Bologna, Italy; 4 Unità Operativa Semplice (UOS) Laboratori di Ricerca e Biobanca, UOC Ricerca Clinica e Traslazionale, AORN Santobono Pausilipon, Napoli, Italy

**Keywords:** enteral nutrition, nutritional support, oral nutrition, parenteral nutrition, pediatric allogeneic hematopoietic stem cell transplantation

## Abstract

Deterioration of nutritional status in allogeneic hematopoietic stem cell transplantation (allo-HSCT) recipients is associated with increased morbidity. Enteral nutrition (EN) has been associated with more favorable outcomes than total parenteral nutrition (TPN); however, TPN remains the first-line nutritional source in many pediatric transplant centers because of health providers and caregiver limitations and biases. Oral nutritional (ON) source may help overcome these limitations, but evidence supporting efficacy in allo-HSCT recipients is currently lacking. We retrospectively evaluated the impact of three nutritional source modalities (ON, EN, and parenteral nutrition) on nutritional status in 125 children undergoing allo-HSCT in two Italian pediatric centers. Secondary endpoints included associations between nutrition modality and several allo-HSCT outcomes: time to engraftment, length of hospitalization, and incidence of acute graft-versus-host disease (aGvHD). A total of 41 patients received TPN, 47/125 received ON, and 37/125 received EN. Patients supported by EN experienced a significantly lower body mass index (BMI) decrease compared to others (−0.45; range −2.18 to 3.95; IQR −0.67 to −0.15 kg/m^2^; *p* < 0.05), while a significantly higher decrease occurred in patients who received TPN compared to all others (−1.1; range −9.86 to 0.58; IQR −1.78 to −0.36; *p* < 0.05); ON was associated with an intermediate outcome (−0.91; range −4.09 to 12.29; IQR −1.66 to −0.06). BMI decrease is greater in male patients (*p* < 0.05). The length of hospitalization was strongly correlated with nutritional support (*p* < 0.001), with EN associated with the lowest median time of admission length. Nutrition support modality did not significantly correlate with severe aGvHD in our analysis, nor was there correlation with time to neutrophil or platelet engraftment.

## 1 Introduction

Allogeneic hematopoietic stem cell transplantation (allo-HSCT) represents a cornerstone in the treatment of many malignant and non-malignant hematological conditions and genetic disorders of childhood (1), and several factors can influence the outcome, including nutritional status (2).

Malnutrition affects 10%–50% of children undergoing allo-HSCT, and it has a complex and multifactorial nature (3). Side effects of the conditioning regimen, especially vomiting, anorexia, diarrhea, and mucositis, as well as gastrointestinal acute graft-versus-host disease (aGvHD), infections, and their associated treatments, have been correlated with decreased oral feeding in allo-HSCT recipients (2). Moreover, conditioning chemotherapy and inflammation status cause an increase in basal metabolic rate and nutritional requirement (4). This combination may result in a severe deterioration of nutritional status, which is associated with increased morbidity and mortality in allo-HSCT (5–8).

For these reasons nutritional sources, including oral (ON) supplements, total parenteral (TPN) and enteral (EN) nutrition, become to optimize for these patients (9–11). TPN and EN provided by a nasogastric tube (NGT) are the two main sources adopted in the transplantation setting in order to provide adequate caloric intake when ON is insufficient (10). Given the association with infectious and metabolic complications, as well as increased direct and indirect healthcare costs, TPN has important limitations. Consequently, EN is currently recommended as the preferred nutritional source for children undergoing allo-HSCT (12, 13). In addition, a recent meta-analysis comparing EN with TPN in allo-HSCT recipients reported significantly lower incidences of aGvHD, including grade III–IV and intestinal aGvHD, in patients who received EN (8).

EN represents a more physiologic feeding approach than TPN, preserving mucosal gut integrity and promoting mucosal repair (14). Despite this, reluctance to use EN as nutritional support is widespread and TPN is still considered the first-line nutritional support in many transplant centers (15–19). The most commonly reported barriers to the use of EN can be perceived poor feasibility and tolerance, NGT-related discomfort, bleeding risk, and refusal of patient or caregiver. Additional obstacles can be related to healthcare providers, such as limited awareness and limited experience, and organizational factors, such as inadequate collaboration and resources (16). Similarly, in the pediatric setting, the most common obstacle was the caregiver’s perception that EN was invasive and painful (17). Given this, one possible solution to overcome these obstacles could be with ON support, as it may be perceived as less invasive and less uncomfortable by both caregivers and physicians. However, evidence supporting that ON support is adequate in allo-HSCT recipients is lacking. In fact, several studies show that oral intake is inadequate in most of allo-HSCT recipients (20); similarly, most studies discuss between EN and TPN support in allo-HSCT recipients since oral intake has already been considered inadequate (8).

In this retrospective study, we evaluate the impact of three nutritional source modalities (ON, EN, and parenteral nutrition, respectively) on nutritional status in children undergoing allo-HSCT. To the best of our knowledge, this is the first report to evaluate the suitability of oral nutritional source in a pediatric allo-HSCT setting.

## 2 Methods

This is a retrospective, multi-center cohort study evaluating the impact of three nutritional support modalities (ON, EN, and parenteral nutrition) on nutritional status in children undergoing allo-HSCT. Secondary endpoints included associations with other allo-HSCT outcomes such as time to engraftment, length of hospital admission, and incidence of severe (grade III–IV) aGvHD.

Data were collected through a chart review of patients who underwent allo-HSCT at the Department of Oncology, Hematology and Cell Therapy of Santobono Pausilipon Children Hospital (Naples, Italy) between 1 January 2014 and 31 December 2023 and at the Department of Pediatric Oncology/Hematology of Policlinico Sant’Orsola (Bologna, Italy) between 1 January 2020 and 31 December 2023. All patients received myeloablative conditioning.

For this analysis, we included patients who were alive at +30 days from HSCT that received only one modality of nutritional support: ON (neutropenic diet with oral caloric supplements), EN by NGT, or TPN. A patient was considered exclusively supported by one modality if >80% of caloric intake was provided by such modality. Neutropenic diet eliminates all raw or undercooked meat, fish and eggs, fresh fruits and juices, fresh vegetables, and unpasteurized dairy products. The compound used for TPN did not have a standardized composition, but it was prescribed individually. The concentration of the given glucose varied from 5.0% to 12.5%. Calories derived from glucose represented 70% of the non-protein calories. The remaining 30% were derived from lipids given in the form of soybean oil emulsion. Proteins were given at a dose of 1 g/kg/day. Oligoelements, vitamins, and electrolytes were also administered as supplements. A commercial formula was utilized for EN. Information regarding the enteral mixture administered is reported in **Supplementary Table 1**.

The following data were collected: sex, age at allo-HSCT, indication for allo-HSCT (malignancy or not), conditioning regimen, donor source, weight before and after allo-HSCT, body mass index (BMI) before and after allo-HSCT, length of hospital stay, time to neutrophil and platelet engraftment, and incidence of grade III–IV aGVHD.

Anthropometric measurements were collected at admission and at day +30 from allo-HSCT. Time to neutrophil and platelet engraftment was defined as the first of three consecutive days with an absolute neutrophil count (ANC) >500/mm³ and a platelet count >20,000/mm³ without transfusion support, respectively. GvHD was defined and graded according to MAGIC criteria (21).

### 2.1 Statistical methods

Data were analyzed using both descriptive and inferential statistics. Continuous variables were summarized as medians with interquartile ranges (IQRs), while categorical variables were reported as counts and percentages. Associations between categorical variables were assessed using the chi-square test or Fisher’s exact test, depending on sample size. The Mann–Whitney *U* test and the Kruskal–Wallis test were used to assess associations between nutrition modalities and continuous variables. The relationship between two continuous variables was tested using Spearman’s rank correlation coefficient.

All statistical analyses were performed using the Easy R software (22). A *p*-value less than or equal to 0.05 was considered statistically significant.

### 2.2 Ethics

Data were anonymized by removing all identifying information and patient details. The study was conducted in accordance with the current approved international guidelines and regulations, and in accordance with the Declaration of Helsinki. Written informed consent was obtained from all patients or their legal guardians.

## 3 Results

### 3.1 Patients

A total of 173 patients underwent allo-HSCT during the study period. Forty-eight patients were excluded from the analysis because anthropometric measurements at established time points were lacking, or because they did not receive a single, exclusive modality of nutritional source. Of the 125 patients included (77 received allo-HSCT at Santobono Pausilipon and 48 received allo-HSCT at Policlinico Sant’Orsola), 124 and 123 were evaluable for neutrophil and platelet engraftment, respectively; the remaining patients were excluded due to graft failure. Length of hospital stay was evaluable in 100 patients because of missing data or early death (**Supplementary Figure 1**).

Patients’ characteristics are summarized in **Table 1**.

**Table 1:** Patients’ characteristics.

	Overall (N = 125)	Total parenteral nutrition (N = 41)	Oral nutrition (N = 47)	Enteral nutrition (NGT) (N = 37)	p-value
Median age at allo-HSCT [range, IQR], years	8.8 [0.3–20.7; 4.3–13.1]	10.1 [0.8–18.6; 4.5–13.7]	8.4 [0.3–18; 3.5–12.8]	8.5 [3.1–20.7; 4.7–12.5]	*p* = 0.67*
Sex:					
Male (%)	83 (66%)	23 (56%)	33 (70%)	27 (74%)	*p* = 0.234^
Female (%)	42 (34%)	18 (44%)	14 (30%)	10 (26%)	
Disease:					
Malignant (%)	93 (74%)	31 (76%)	30 (64%)	31 (83%)	*p* = 0.12^
Not malignant (%)	33 (26%)	10 (24%)	17 (36%)	6 (16%)	
HSC source:					
PBSC (%)	36 (29%)	8 (20%)	16 (34%)	12 (32%)	*p* = 0.349^
BM (%)	85 (68%)	31 (76%)	29 (62%)	25 (68%)	
CB (%)	4 (3%)	2 (5%)	2 (4%)	0	
Donor:					
Sibling (%)	26 (21%)	6 (15%)	12 (26%)	8 (22%)	*p* = 0.534^
MUD (%)	58 (46%)	22 (54%)	20 (43%)	16 (43%)	
UCB (%)	4 (3%)	2 (5%)	2 (3%)	0	
Haploidentical (%)	35 (28%)	11 (27%)	11 (23%)	13 (35%)	
HLA phenotypically identical (%)	2 (2%)	0	2 (3%)	0	
Severe mucositis (%)	32 (26%)	15 (37%)	10 (21%)	7 (19%)	*p* = 0.163^
Median weight before allo-HSCT [range, IQR], kg	28 [5.5–85; 16–48.5]	27.3 [5.5–85; 16–54]	28 [5.52–76.7; 14.25–43.975]	30 [13–90; 19–58.4]	*p* = 0.485*
Median weight after allo-HSCT [range, IQR], kg	25.5 [5.5–79.7; 16.1–45.4]	24.3 [5.45–79.7; 15.7–49]	26.3 [5.6–72.5; 14.4–41.1]	28 [12.8–77.5; 18.4–50.3]	*p* = 0.429*
Median BMI before allo-HSCT [range, IQR]	17.1 [6–33.7; 15.3–20.4]	16.9 [6–33.7; 14.7– 21.1]	17.3 [8–27.1; 15.4–20]	17.6 [12.7–27; 15.7–20]	*p* = 0.912*
Median BMI after allo-HSCT [range, IQR]	16.7 [6–32.6;14.6–19.1]	16.3 [5.6–32.6; 13.9–19.1]	17.2 [12.6–26.8; 15.3–18.9]	17 [13.2–27.2; 14.8–19.6]	*p* = 0.435*

BM, bone marrow; BMI, body mass index; CB, cord blood; HSC, hematopoietic stem cell; HSCT, hematopoietic stem cell transplantation; IQR, interquartile range; MUD, matched unrelated donor; NGT, nasogastric tube; PBSC, peripheral blood stem cell; UCB, unrelated cordblood; *Kruskal–Wallis test; ^ chi-square test.

Eighty-three patients (66%) were male and 42 (34%) were female. The median age at allo-HSCT was 8.8 years (range 0.3–20.7; IQR 4.3–13.1 years). Underlying diseases were malignant in 93 patients (74%) and non-malignant in 33 patients (26%). A total of 41 patients received TPN for a median time of 14 days (range 5–44, IQR 11–21), and 37 of 125 received EN for a median time of 20 days (range 7–34, IQR 14.5–26.5). A total of 47 patients received ON with nutrient-dense supplements. Age, sex, underlying disease, donor source, and hematopoietic stem cell source did not differ significantly among the three subgroups (**Table 1**).

### 3.2 Anthropometric outcomes

**Figure 1:**
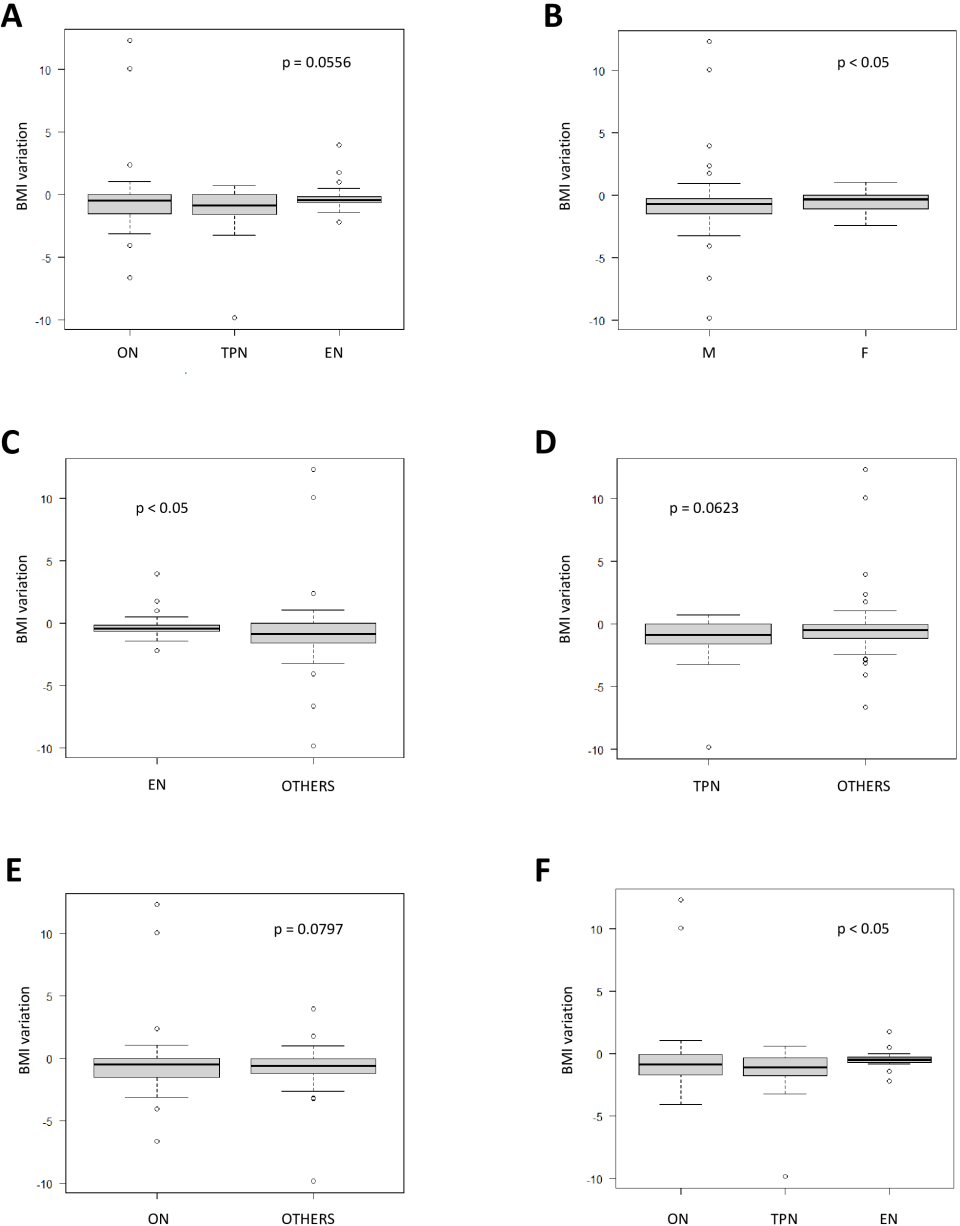
**(A)** BMI variation according to nutrition support (Kruskal–Wallis test). **(B)** BMI variation according to gender (Mann–Whitney *U* test). **(C)** Comparison of BMI variation between EN and other modalities (Mann–Whitney *U* test). **(D)** Comparison of BMI variation between TPN and other modalities (Mann–Whitney *U* test). **(E)** Comparison of BMI variation between EN and other modalities (Mann–Whitney *U* test). **(F)** Analysis of BMI variation according to nutrition support in patients who underwent allo-HSCT because of malignant disease (Kruskal–Wallis test). BMI, body mass index; EN, enteral nutrition; F, female; HSCT, hematopoietic stem cell transplantation; M, male; ON, oral nutrition; TPN, totalparenteral nutrition.

Median weight and BMI before transplantation were 28 kg (range 5.5–85; IQR 16–48.5 kg) and 17.1 kg/m^2^ (range 6–33.7; IQR 15.3–20.4 kg/m^2^), respectively, with no significant differences across subgroups (**Table 1**). A median decrease of −0.6 kg/m^2^ (range −9.86 to 12.29; IQR −1.34 to −0.04 kg/m^2^) in BMI was observed across all groups after allo-HSCT (**Table 2**); BMI decrease was greater in male patients (*p* < 0.05; **Supplementary Table 2** and **Figure 1**) and was directly proportional to age (*p* = 0.0017). Patients supported by EN experienced a significantly lower decrease compared with the other groups (−0.45; range −2.18 to 3.95; IQR −0.67 to −0.15 kg/m^2^; *p* < 0.05; **Figure 1**), while a significantly higher decrease occurred in patients who received TPN compared to all others (−1.1; range −9.86 to 0.58; IQR −1.78 to −0.36; *p* < 0.05).

**Table 2:** BMI variation and weight loss across all subgroups.

	Overall (*N* = 125)	Total parenteral nutrition (*N* = 41)	Oral nutrition (*N* = 47)	Enteral nutrition (NGT) (*N* = 37)	*p*-value
Median BMI variation after allo-HSCT [range, IQR]	−0.6 [−9.86 to 12.29; −1.34 to −0.04]	−0.9 [−9.86 to 0.73; −1.61 to 0]	−0.49 [−6.65 to 12.29; −1.52 to −0.01]	−0.45 [−2.18 to 3.95; −0.67 to −0.15]	*p* = 0.0556*
Median weight change after allo-HSCT [range, IQR], %	−3% [−36% to +69%; −6% to −1%]	−5% [−36% to +4%; −9% to 0]	−3% [−26% to +69%; −7.5% to 0]	−2% [−8% to +17%; −3% to −1%]	*p* = 0.0165*

BMI, body mass index; HSCT, hematopoietic stem cell transplantation; IQR, interquartile range; NGT, nasogastric tube; * Kruskal–Wallis test.

Interestingly, ON resulted in an intermediate outcome (−0.91; range −4.09 to 12.29; IQR −1.66 to −0.06), which was neither significantly worse than EN (*p* = 0.0785) nor significantly better than TPN (*p* = 0.514).

As expected, BMI decrease was closely correlated with weight loss, with a median percentage of weight change of −3% (range from −36% to +69%; IQR −6% to −1%); male patients and patients fed with TPN experienced a higher loss compared to the others, with a median decrease of −3% (range −36% to +69%; IQR −8% to −1%; *p* < 0.05) and −5% (range −36% to +4%; IQR −9% to 0; *p* < 0.05), respectively (**Table 2**, **Supplementary Figure 2**, and **Supplementary Table 2**).

Restricting the analysis to patients undergoing allo-HSCT due to malignant disease, we confirm that BMI variation and weight loss significantly depend on nutritional source modality. In detail, children supported by EN experienced a significantly lower median BMI decrease compared with the other groups (−0.48; range −2.18 to 1.76; IQR −0.69 to −0.28 kg/m^2^; *p* < 0.05), while a significantly higher decrease affected patients receiving TPN compared to all others (−1.1; range −9.86 to 0.58; IQR −1.78 to −0.36; *p* < 0.05). Similarly, EN resulted in a lower median percentage weight loss compared to others (−2%; range −8% to +17%; IQR −3% to −1%; *p* < 0.05), while children supported by TPN suffered from a higher loss compared to others (−6%; range −36% to +4%; IQR −9% to −1.5%; *p* < 0.05). We also analyzed BMI variation and weight loss by stratifying by age group: 0–1 year, 1–3 years, and >3 years. Where a reliable analysis was not possible for the two younger age groups due to the small number of patients, the results in patients >3 years of age are consistent with those obtained in the general population (see **Supplementary Table 3** for details).

See also **Figure 1**, **Supplementary Figure 2**, and **Table 3**.

**Table 3:** Median BMI variation and weight loss in patients underwent allo-HSCT because of malignant disease.

	Overall (*N* = 92)	Total parenteral nutrition (*N* = 31)	Oral nutrition (*N* = 30)	Enteral nutrition (NGT) (*N* = 31)	*p*-value
Median BMI variation after allo-HSCT [range, IQR]	−0.69 [−9.86 to 12.29; −1.44 to −0.18]	−1.1 [−9.86 to 0.58; −1.78 to −0.36]	−0.91 [−4.09 to 12.29; −1.66 to −0.06]	−0.48 [−2.18 to 1.76; −0.69 to −0.28]	*p* = 0.0271*
Median weight change after allo-HSCT [range, IQR], %	−3% [−36% to + 69%; −7% to −1%]	−6% [−36% to +4%; −9% to −1.5%]	−5% [−16% to +69%; −8.75% to −0.25%]	−2% [−8% to +17%; −3% to −1%]	*p* = 0.0131*

BMI, body mass index; HSCT, hematopoietic stem cell transplantation; IQR, interquartile range; NGT, nasogastric tube; * Kruskal–Wallis test.

### 3.3 Transplant outcomes

We found that hospitalization duration was significantly correlated with nutritional source (*p* < 0.001). In particular, EN was associated with the shortest median time of hospitalization (35 days, range 25−193, IQR 30−44), while TPN was associated with the longest (55 days, range 32–135, IQR 44–70); patients supported by ON remain hospitalized for a median time of 42 days (range 27–147, IQR 36–53; see also **Supplementary Table 4**). Interestingly, ON was associated with significantly shorter hospitalization compared to TPN (*p* < 0.001), although the length of hospitalization remains longer in patients who received ON compared to EN (*p* < 0.05).

Severe (namely, grade III–IV) aGVHD occurred in 6/47 patients who underwent ON, in 5/41 patients fed with TPN, and in 7/37 children who received EN, respectively; nutrition support modality did not significantly correlate with severe aGvHD in our analysis (*p* = 0.691; **Supplementary Table 5**). Severe gastrointestinal aGvHD occurred in 4/47 children supported by ON, in 3/41 children who received TPN, and in 3/37 children supported by EN (*p* = 1).

Similarly, nutritional modality did not significantly correlate with time to neutrophil nor platelet engraftment (**Supplementary Table 6** and **Supplementary Table S7**).

Severe (namely, grade 3–4 according to CTCAE 4.0) mucositis occurred in 10/47 children in the ON group, in 15/41 patients supported by TPN, and in 7/37 children fed with EN, respectively. The incidence did not differ significantly between the three subgroups (*p* = 0.163; **Table 1**).

## 4 Discussion

This retrospective study that was conducted in two pediatric centers performing allo-HSCT compared, for the first time, three different nutritional sources in children undergoing allo-HSCT: ON, EN, and TPN. Our study showed that EN was associated with maintained nutritional status in terms of significantly less weight loss and maintained BMI compared to TPN, in the early phase post-transplant period, when a number of factors related to conditioning regimens, the catabolic effect of infections, or malnutrition can induce loss of muscle mass. To our knowledge, no other studies demonstrated a clear advantage of EN over TPN in preventing BMI or weight loss in pediatric allo-HSCT recipients (8, 23, 24). Consistently, a pilot study in autologous HSCT recipients found no significant differences in body composition parameters between patients receiving EN and those receiving TPN at multiple time points post-transplantation (25).

The majority of studies conducted in the allo-HSCT setting are more focused on transplant-related clinical outcomes rather than anthropometric measurements. All the studies conducted exclusively compare EN with TPN, due to the very limited use of ON among pediatric patients. ON is generally considered inadequate in most allo-HSCT recipients because of several reasons including food refusal or uncontrolled pain (8, 20). Zama and colleagues observed no statistically significant difference between EN and other groups regarding BMI and weight loss during hospitalization (26). Similarly, Guièze et al. comparing EN with TPN in 56 patients reported no significant differences in weight or BMI (27). No significant differences in body weight between patients supported by EN versus those fed with TPN was also found in the prospective study by Seguy et al. (28).

It is important to note that in our study, 47 patients received ON, made possible by an effective pain control program to mitigate mucositis and by the provision of calorie-dense, palatable foods selected according to the patients’ preferences. In our study, ON did not significantly differ from EN or TPN regarding BMI and weight loss, supporting the idea that it can provide sufficient nutritional support without exposing patients to TPN adverse effects when EN is not feasible.

Our study showed that children supported by EN had a shorter length of hospitalization compared with those receiving TPN, in line with the findings reported by Gonzales et al. and Zemrani et al. (24, 29). Conversely, other studies failed to demonstrate a clear superiority of EN over TPN (26–28, 30–33), resulting in the continuing debate on the topic within the scientific community.

Regarding the transplant outcomes, in our retrospective cohort, nutrition source did not appear to influence the occurrence of severe aGvHD in contrast with the results of a recent meta-analysis published by Zama and colleagues (8), which analyzed the results of five studies including a total of 522 patients (24, 27, 28, 33, 34), showing a significant protective effect of EN over TPN from developing aGvHD grade III–IV. More recent studies are in line with this result (35). The limited sample size compared to the cited studies may explain the discrepancy between our results and the scientific literature.

In our report, the time to platelet engraftment was not correlated with nutritional modality, consistent with the results of Skaarud et al. and Guièze and colleagues (27, 33). In contrast, Gonzales and colleagues observed a shorter time to platelet engraftment in patients receiving exclusively EN (24). Similarly, in their study, Seguy et al. reported a shorter time to platelet engraftment in the EN group compared to the TPN group, but the result was not confirmed by a multivariate analysis (28). On the other hand, Zama and colleagues observed an earlier platelet engraftment in children who received TPN when considering a platelet count threshold of >20 × 10^9^/L but not at a >50 × 10^9^/L threshold (26). No difference in time to neutrophil engraftment among ON, EN, or TPN was found in our report, consistent with the results of most studies (24, 26, 27, 33), whereas Seguy and colleagues observed a shorter time in the EN group compared to TPN (28).

In this study, all patients received a neutropenic ON diet. Despite the fact that several studies and meta-analyses showed no benefit over a liberalized diet in preventing foodborne infections (36, 37), the neutropenic diet is still adopted in most pediatric transplant centers (38–40). Such dietary restrictions may have affected diet palatability and potentially compromised dietary intake, although recent studies have failed to demonstrate an increase in calorie intake with a liberalized diet (41, 42).

Our study has several limitations. First, the retrospective design and the small cohort size limit the generalization of the results, since unrecognized bias may have influenced the results: for example, patients in better overall clinical condition may have been more likely to adhere to adequate ON intake and refuse EN and TPN nutritional sources. In addition, BMI and weight loss are not the best surrogate for nutritional status in the pediatric population because, although easily applicable in clinical practice, they lack information on body composition and are heavily influenced by hydro-electrolytic imbalance occurring during the allo-HSCT (11). Moreover, we considered in the analysis only patients exclusively supported by one modality, whereas a combination of the three approaches may be beneficial, especially with the aim of reducing the use of PB by combining it with EN or ON (13). The long study period may have influenced outcomes due to advances in allo-HSCT technologies and supportive care. Finally, the different institutional practices among the two centers involved may affect length of hospital admission.

In conclusion, our study confirms recent evidence supporting the preferential use of EN over TPN in nutritional source for pediatric allo-HSCT recipients. It also supports the idea that ON can be a valid nutritional support, when patients are in good clinical condition, complemented by an adequate pain control program to mitigate the effects of mucositis and support form a dietician that can help provide adequate calorie intake concerning palatable food that kids prefer. Prospective randomized studies are warranted to investigate the role of ON alone in the nutritional support of children undergoing allo-HSCT and to confirm its non-inferiority compared to TPN.

## Data Availability

The raw data supporting the conclusions of this article will be made available by the authors, without undue reservation.
