# Supplementary material for: Impact of nutritional source modality on weight loss and BMI reduction after hematopoietic stem cell transplantation
Source: Front Oncol. 2026 Feb 25;16:1778224. doi: 10.3389/fonc.2026.1778224 (PMC12975454; doi:10.3389/fonc.2026.1778224)
Supplement: Supplementary file 1 [file DataSheet1.docx]

Supplementary Material

# Supplementary Tables

**Supplemental Table S1.** The commercial formula used for EN.

| Nutritional Information for 100 g of product: | | |
| --- | --- | --- |
| Energy | | 1992 kJ |
|  |  | 475 kcal |
| Fat (42 En%): | | 22 g |
|  | Saturated | 9.4 g |
|  | Monosaturated | 7.7 g |
|  | Polysaturated | 4.0 g |
| Carbohydrates (47 En%): | | 56 g |
|  | Sugars | 5.0 g |
| Dietary Fiber (0 En%) | | 0 g |
| Proteins (11 En%) | | 13.3 g |
| Salt | | 0.71 g |
| Vitamins | |  |
|  | Vit.A | 214 µg |
|  | Vit.D | 6.2 µg |
|  | Vit.E | 6.7 mg |
|  | Vit.K | 19 mg |
|  | Thiamine | 0.48 mg |
|  | Riboflavin | 0.95 mg |
|  | Niacin | 5.3 mg |
|  | Pantothenic acid | 1.9 mg |
|  | Vit.B_6_ | 0.48 mg |
|  | Folate | 71.3 µg |
|  | Vit.B_12_ | 1.2 µg |
|  | Biotin | 14.3 µg |
|  | Vit.C | 47.5 mg |
| Minerals | | |
|  | Na | 285 mg |
|  | K | 546 mg |
|  | Cl | 437 mg |
|  | Ca | 428 mg |
|  | P | 309 mg |
|  | Mg | 66.5 mg |
|  | Fe | 5.7 mg |
|  | Zn | 5.2 mg |
|  | Cu | 0.48 mg |
|  | Mn | 0.29 mg |
|  | Mo | 24.4 µg |
|  | Se | 15.2 µg |
|  | Cr | 7.6 µg |
|  | I | 71.4 µg |
| Others | | |
|  | Choline | 95.0 mg |
|  | Inositol | 26.1 mg |
|  | L-carnitine | 15.8 mg |
|  | Taurine | 31.5 mg |

**Supplemental Table S2.** BMI variation and weight loss according to gender. BMI = Body mass index; HSCT = Hematopoietic stem cell transplantation; IQR = interquartile range; * Mann Whitney U test.

|  | **Overall**  **(N=125)** | **Male**  **(N=83)** | **Female**  **(N=42)** | **P-value** |
| --- | --- | --- | --- | --- |
| Median BMI variation after allo-HSCT  [range, IQR] | -0.6  [-9.86 to 12.29; -1.34 to -0.04] | -0.71  [-9.86 to +12.29; -1.49 to -0.29] | -0.34  [-2.4 to +1.02; -1.10 to +0.1] | p = 0.0453* |
| Median weight change after allo-HSCT  [range, IQR], % | -3%  [-36% to +69%; -6% to -1%] | -3%  [-36% to +69%; -8% to -1%] | -2%  [-11% to+24%; -5% to 0] | p = 0.0161* |

**Supplemental Table S3.** BMI variation and weight loss stratified for age. BMI = Body mass index; HSCT = Hematopoietic stem cell transplantation; IQR = interquartile range; NGT = nasogastric tube * Kruskal Wallis test.

| **Age < 1 years** | | | | |
| --- | --- | --- | --- | --- |
|  | **Total parenteral nutrition**  **(N=2)** | **Oral nutrition**  **(N=6)** | **Enteral nutrition (NGT)**  **(N=0)** | **P-value** |
| Median BMI variation after allo-HSCT  [range, IQR] | -0.2  [-3.27 to 0.55; -0.79 to 0] | -0.33  [-1.86 to 2.37; -0.91 to 0.5] | NA | p = 0.772* |
| Median % weight variation after allo-HSCT  [range, IQR], % | -6%  [-12% to 0; -9% to -3%] | +3%  [-3% to 13%; 0.25% to 5.75%] | NA | p = 0.131* |
| **Age 1-3 years** | | | | |
|  | **Total parenteral nutrition**  **(N=6)** | **Oral nutrition**  **(N=4)** | **Enteral nutrition (NGT)**  **(N=0)** | **P-value** |
| Median BMI variation after allo-HSCT  [range, IQR] | -0.31  [-0.9 to 0.55; -0.73 to 0] | -0.37  [-1.86 to 0.3; -0.74 to -0.35] | NA | p = 0.521* |
| Median % weight variation after allo-HSCT  [range, IQR], % | -2.5%  [-6% to +3%; -5% to 0] | -2%  [-2% to -2%; -2% to -2%;] | NA | p = 1* |
| **Age > 3 years** | | | | |
|  | **Total parenteral nutrition**  **(N=33)** | **Oral nutrition**  **(N=37)** | **Enteral nutrition (NGT)**  **(N=37)** | **P-value** |
| Median BMI variation after allo-HSCT  [range, IQR] | -1.11  [--9.86 to 0.73; -1.72 to -0.34] | -0.9  [-6.65 to 12.29; -1.7 to -0.04] | -0.45  [-2.18 to 3.95; -0.67 to -0.15] | p = 0.0145* |
| Median % weight variation after allo-HSCT  [range, IQR], % | -6%  [-36% to +4%; -9% to -2%] | -6%  [-26% to +69%; -9% to -1%] | -2%  [-8% to +17%; -3% to -1%] | p = 0.00204* |

**Supplemental Table S4.** Median length of admission after HSCT according to nutritional support. HSCT = Hematopoietic stem cell transplantation; IQR = interquartile range; NGT = nasogastric tube; * Kruskal-Wallis test.

|  | **Overall**  **(N=100)** | **Total parenteral nutrition**  **(N=32)** | **Oral nutrition**  **(N=47)** | **Enteral nutrition (NGT)**  **(N=21)** | **P-value** |
| --- | --- | --- | --- | --- | --- |
| Median length of stay after allo-HSCT  [range, IQR], days | 43  [25-193; 35.25-58] | 55  [32-135; 43.75-69.5] | 42  [27-147; 36-52.5] | 35  [25-193; 30-44] | p = 0.000037* |

**Supplemental Table S5.** Grade III-IV aGvHD occurrence according to nutritional support. aGvHD = acute graft-versus-host disease; NGT = nasogastric tube; * Fisher's Exact Test.

| **Severe aGVHD** | **Overall**  **(N=125)** | **Total parenteral nutrition**  **(N=41)** | **Oral nutrition**  **(N=47)** | **Enteral nutrition (NGT)**  **(N=37)** | **P-value** |
| --- | --- | --- | --- | --- | --- |
| Yes | 18 | 5 | 6 | 7 | p = 0.691* |
| No | 107 | 36 | 41 | 30 |  |

**Supplemental Table S6.** Median time to neutrophils’ engraftment according to nutritional support. HSCT = Hematopoietic stem cell transplantation; IQR = interquartile range; NGT = nasogastric tube; * Kruskal-Wallis test.

|  | **Overall**  **(N=124)** | **Total parenteral nutrition**  **(N=41)** | **Oral nutrition**  **(N=46)** | **Enteral nutrition (NGT)**  **(N=37)** | **P-value** |
| --- | --- | --- | --- | --- | --- |
| Median time to neutrophils’ engraftment  [range, IQR], days | 19  [8-45; 15-22] | 19  [8-45; 15-22] | 19.5  [11-34; 15.25-22.75] | 18  [11-32; 14-20] | p = 0.203* |

**Supplemental Table S7.** Median time to platelets’ engraftment according to nutritional support. HSCT = Hematopoietic stem cell transplantation; IQR = interquartile range; NGT = nasogastric tube; * Kruskal-Wallis test.

|  | **Overall**  **(N=123)** | **Total parenteral nutrition**  **(N=40)** | **Oral nutrition**  **(N=46)** | **Enteral nutrition (NGT)**  **(N=37)** | **P-value** |
| --- | --- | --- | --- | --- | --- |
| Median time to platelets’ engraftment  [range, IQR], days | 20  [11-70; 16-26] | 20  [11-70; 16-30] | 21  [11-61; 16-25] | 19  [12-44; 16-23] | p = 0.634* |

## Supplementary Figures

**Supplementary Figure S1.** Flow diagram according to STROBE checklist.

**Supplementary Figure S2.** (A) Percentage of weight loss variation according to nutrition support (Kruskal-Wallis test). (B) Weight loss according to gender (Mann Whitney U test). (C) Comparation of weight loss between EN and other modalities (Mann Whitney U test). (D) Comparation of weight loss between TPN and other modalities (Mann Whitney U test). (E) Comparation of weight loss between EN and other modalities (Mann Whitney U test). (F): Analysis of weight loss according to nutrition support in patients underwent HSCT because of malignant disease (Kruskal-Wallis test). EN = enteral nutrition; F = female; HSCT = Hematopoietic stem cell transplantation; M = male; ON = oral nutrition; TPN = total parenteral nutrition.
