# Supplementary figures and images for: Impact of nutritional source modality on weight loss and BMI reduction after hematopoietic stem cell transplantation
Source: Front Oncol. 2026 Feb 25;16:1778224. doi: 10.3389/fonc.2026.1778224 (PMC12975454; doi:10.3389/fonc.2026.1778224)

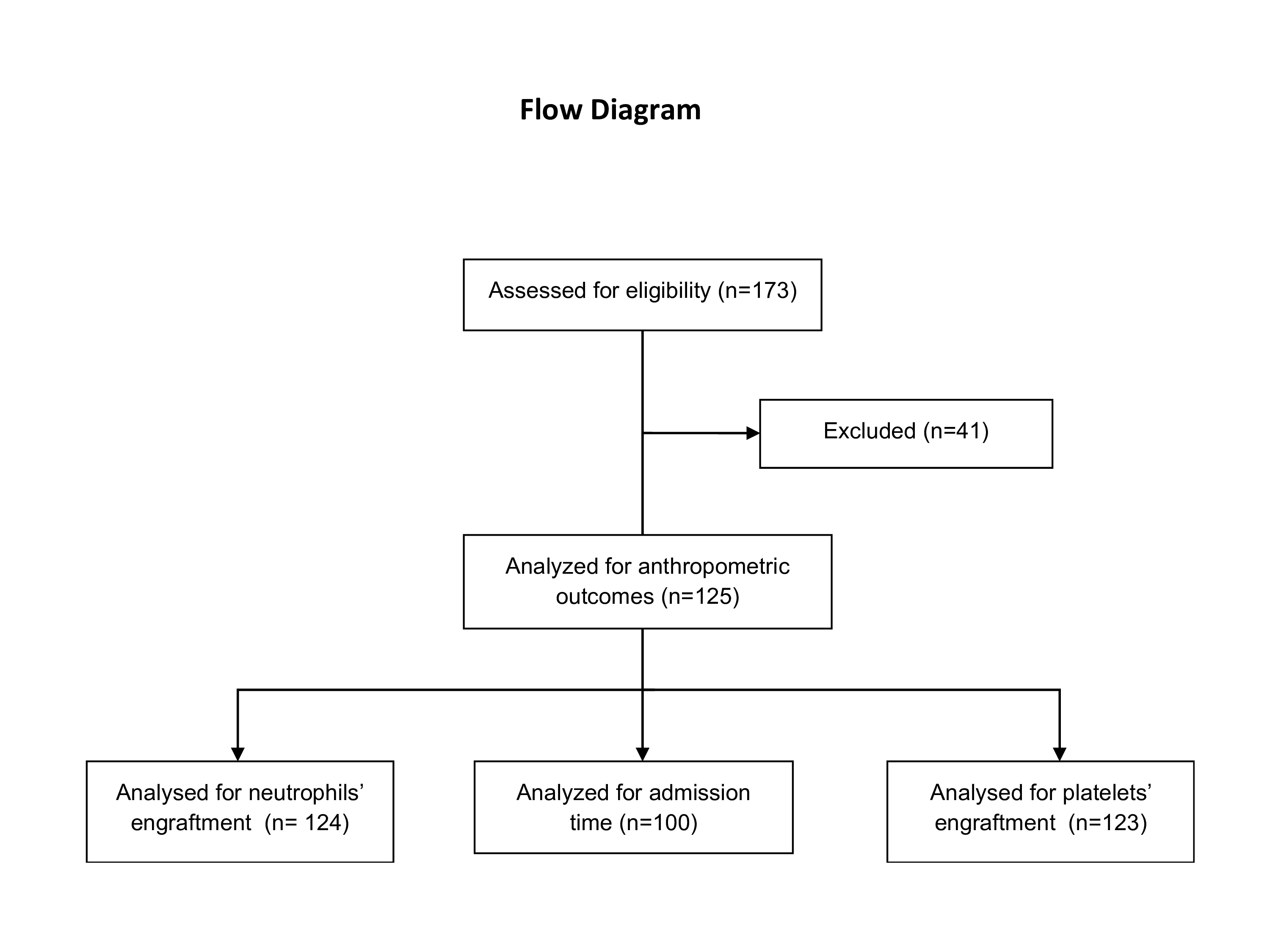

Supplement: Supplementary file 2 [file Image1.tif]

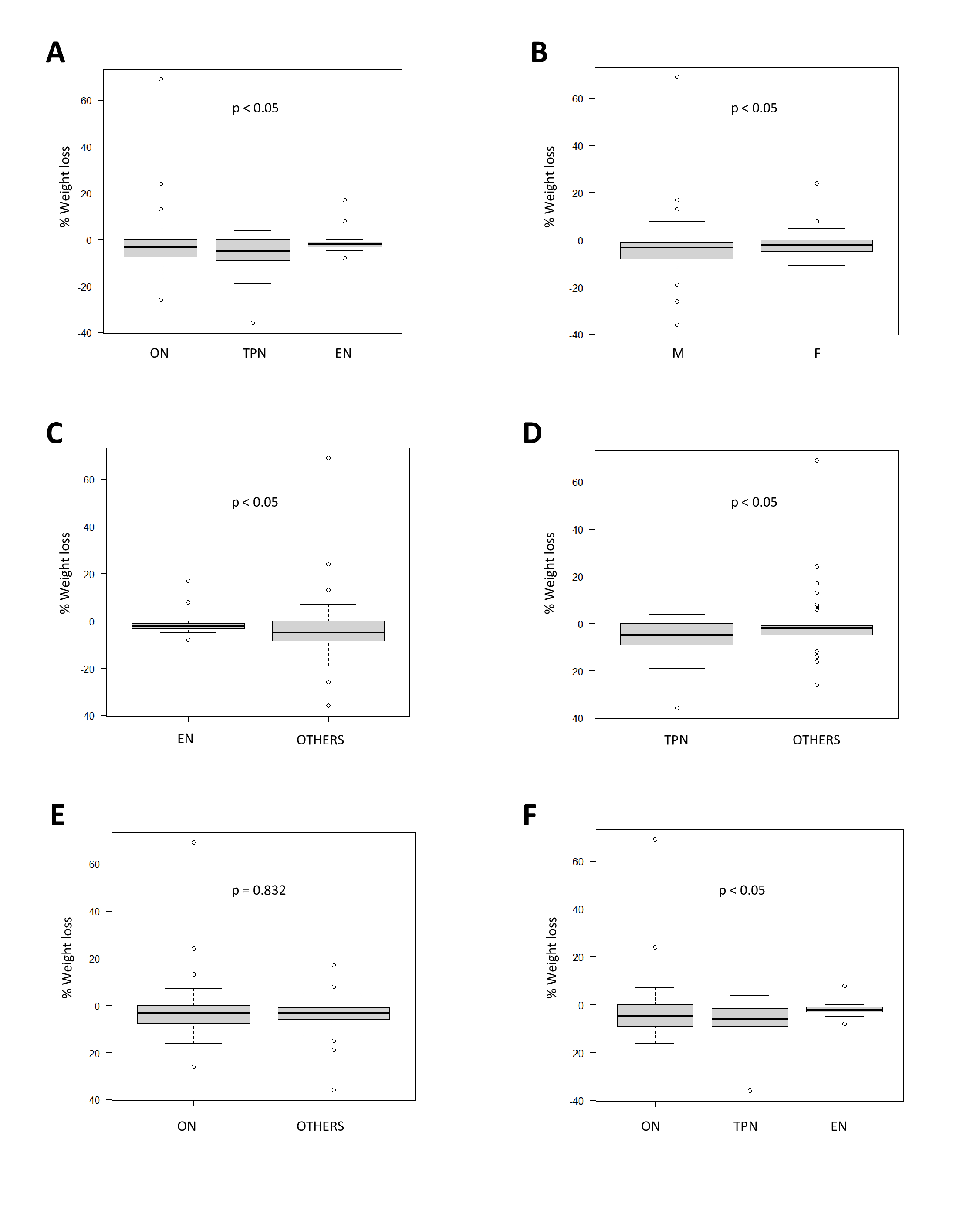

Supplement: Supplementary file 3 [file Image2.tif]
